# The PWWP2A Histone Deacetylase Complex Represses Intragenic Spurious Transcription Initiation in mESCs

**DOI:** 10.1016/j.isci.2020.101741

**Published:** 2020-10-29

**Authors:** Guifeng Wei, Neil Brockdorff, Tianyi Zhang

**Affiliations:** 1 Development Epigenetics, Department of Biochemistry, University of Oxford, Oxford OX1 3QU, UK; 2 Lymphocyte Development and Single Molecule Imaging, MRC London Institute of Medical Sciences, Institute of Clinical Sciences, Faculty of Medicine, Imperial College London, London W12 0NN, UK

**Keywords:** Molecular Biology, Molecular Mechanism of Gene Regulation, Stem Cells Research

## Abstract

Transcriptional fidelity depends on accurate promoter selection and initiation from the correct sites. In yeast, H3K36me3-mediated recruitment of the Rpd3S HDAC complex to gene bodies suppresses spurious transcription initiation. Here we describe an equivalent pathway in metazoans. PWWP2A/B is an H3K36me3 reader that forms a stable complex with HDAC1/2. We used CAGE-seq to profile all transcription initiation sites in wild-type mESCs and cells lacking PWWP2A/B. Loss of PWWP2A/B enhances spurious initiation from intragenic sites present in wild-type mESCs, and this effect is associated with increased levels of initiating Pol-II and histone acetylation. Spurious initiation events in *Pwwp2a/b* DKO mESCs do not overlap in genomic location or chromatin features with spurious sites that arise in *Dnmt3b* KO mESCs, previously reported to function in the suppression of intragenic transcriptional initiation, suggesting these pathways function cooperatively in maintaining the fidelity of transcription initiation in metazoans.

## Highlights

• Loss of PWWP2A/B leads to increased levels of spurious transcription initiation
• Spurious TSS sites are predominantly in the gene bodies of highly expressed genes
• Spurious sites are marked with increased histone acetylation and initiating Pol II
• PWWP2-spurious TSSs are distinct from those caused by DNMT3B loss

## Graphical Abstract

**Figure:**
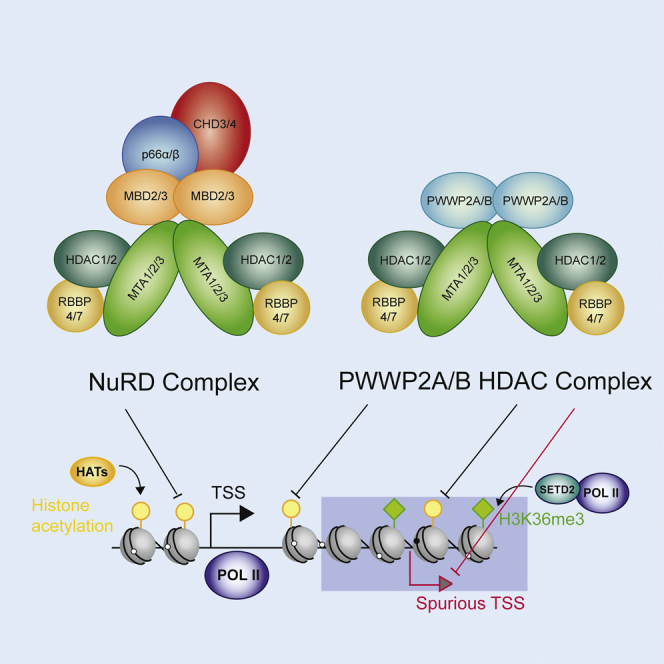

## Introduction

Trimethylation of lysine 36 on histone H3 (H3K36me3) is a highly conserved posttranslational histone modification in eukaryotic organisms and is enriched over the bodies of actively transcribed genes (Bannister et al., 2005). H3K36me3 is recognized by the PWWP domain, a motif that is present in many chromatin-modifying complexes (Dhayalan et al., 2010; Qin and Min, 2014; Tian et al., 2019; van Nuland et al., 2013; Wen et al., 2014; Xu et al., 2008). These readers are involved in a variety of biological processes, including intragenic DNA methylation, transcription elongation, DNA repair, alternative splicing, and repression of spurious transcription initiation (as reviewed in Huang and Zhu, 2018; Wagner and Carpenter, 2012). Furthermore, deregulation of H3K36 methylation and mutations of associated factors are linked to developmental disorders and cancers (as reviewed in Li et al., 2019; Zaghi et al., 2019).

Studies in yeast and mammalian cells have found that H3K36me3 is important for maintaining transcriptional fidelity through repression of spurious transcription initiation from within coding regions. Mechanistically, H3K36me3-mediated recruitment of effectors from two different epigenetic pathways, DNA methylation and histone deacetylation, has been linked to the suppression of spurious intragenic transcription initiation in actively transcribed genes. Human cancer cells treated with inhibitors against DNA methyltransferases and histone deacetylases show elevated levels of spurious transcription initiation (Brocks et al., 2017). Loss of the H3K36me3-binding DNA methyltransferase DNMT3B in mouse embryonic stem cells (mESCs) leads to increased spurious transcription initiation (Neri et al., 2017). In *Saccharomyces cerevisiae*, H3K36me3 recruits the Rpd3S HDAC complex to deacetylate histones across coding regions to repress spurious transcription initiation (Carrozza et al., 2005; Joshi and Struhl, 2005; Keogh et al., 2005), but mammalian equivalents of this pathway remained uncharacterized. In this study, we uncover a newly described PWWP2A/B-HDAC complex in mammalian cells that suppresses transcription initiation from the gene bodies of actively transcribed genes.

**Figure 1:**
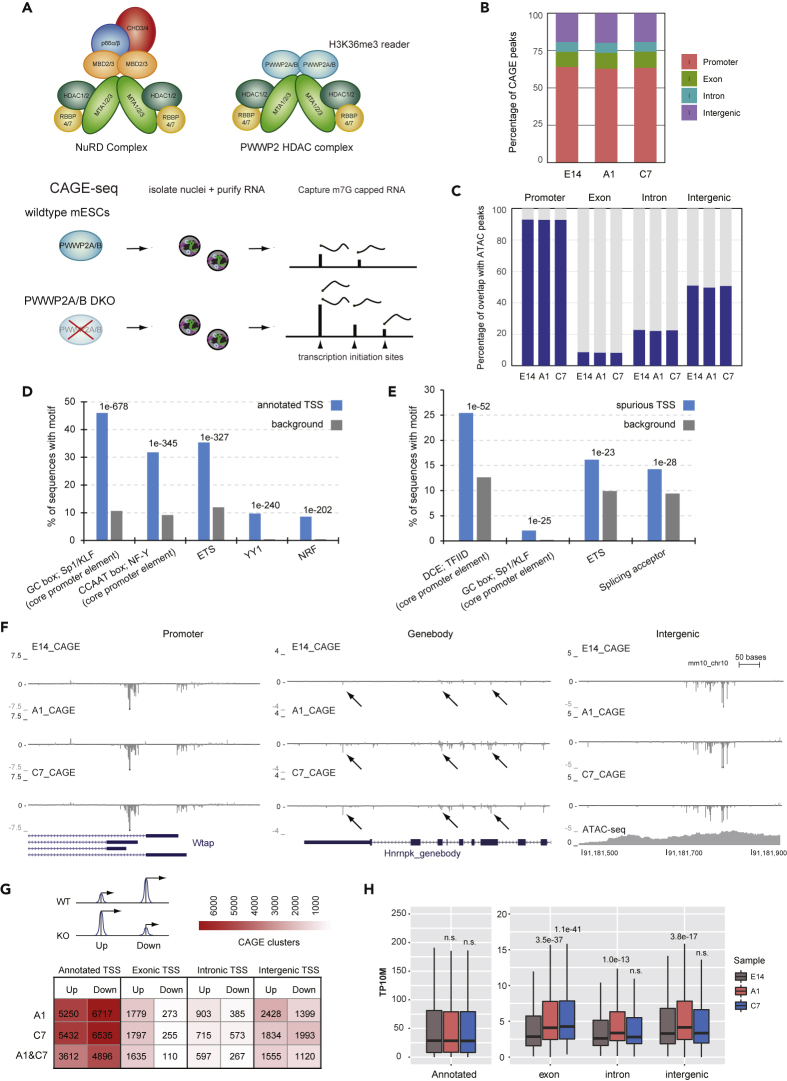
Loss of PWWP2A/B Leads to Increased Levels of intragenic Spurious Transcription Initiation in mESCs (A) Comparison of the NuRD and PWWP2A/B HDAC complex, which contains the H3K36me3-binding PWWP2A/B protein bound to the MTA1/2/3:HDAC1/2:RBBP4/7 HDAC subcomplex (top). Schematic of nuclear RNA CAGE-seq from wild-type E14 and *Pwwp2a/b* DKO mESCs (bottom). (B) The percentage of consensus TSS detected by CAGE-seq in wild-type E14 and *Pwwp2a/b* DKO clones A1 and C7 classified into four groups: annotated promoter, exonic, intronic, or intergenic TSS. See also Table S1. (C) The percentage of CAGE-seq TSSs in each group (annotated promoter, exon, intron, intergenic) that overlap with ATAC-seq peaks for wild-type E14 and *Pwwp2a/b* DKO lines A1 and C7. See also Figure S1. (D) Annotated promoter TSSs (blue bars) detected by CAGE in mESCs are enriched in core promoter elements (GC box, CCAAT box) and in transcription factor motifs commonly found at promoters (ETS, YY1, NRF) compared with randomly generated background sequences (gray bars). The top five most abundant and significant motifs found using Homer are shown with the significance value. (E) Spurious TSSs (blue bars) from wild-type E14 mESCs are slightly enriched in core promoter elements (DCE, GC box) and transcription factor (ETS) sequences compared with randomly generated background sequences (gray bars). The top four most significant motifs found using Homer are shown with the significance value. (F) UCSC genome browser view of the CAGE signal across several sites. Left: CAGE captures TSSs of different isoforms of the *Wtap* gene. Middle: transcription initiation peaks detected in the gene body of the *HnrnpK* gene where arrows indicate increased spurious transcription initiation events in *Pwwp2a/b* DKO lines. Right: intergenic transcription initiation event that overlaps with a region of high chromatin accessibility, possibly an enhancer. See also Figure S2. (G) CAGE-seq expression level heatmap for annotated promoter, exonic, intronic, and intergenic TSSs divided into two groups based on behavior: Up (increased CAGE expression in *Pwwp2a/b* DKO compared with wild-type) and Down (decreased CAGE expression in *Pwwp2a/b* DKO compared with wild-type). More spurious exonic and intronic TSSs go Up than Down in *Pwwp2a/b* DKO cells. The bottom row shows the common events shared between the two clones. (H) CAGE expression for annotated promoter, exonic, intronic, and intergenic TSSs in wild-type E14 and *Pwwp2a/b* DKO lines calculated using the CAGEr package in tags per 10 million (TP10M). The p values between wild-type E14 and DKO lines were calculated using the two-sided Mann-Whitney test. The exonic TSSs shows significantly more transcription initiation in the *Pwwp2a/b* DKO lines.

Recently, we and others have identified a variant nucleosome remodeling and deacetylase (NuRD) complex, in which the histone deacetylase subunits of NuRD (MTA1/2/3,HDAC1/2, RBBP4/7) form a highly stable and stoichiometric complex with the H3K36me3-reader PWWP2A and its paralog PWWP2B (Figure 1A) (Link et al., 2018; Zhang et al., 2018). In this complex, PWWP2A/B, MTA1/2/3, and HDAC1/2 are present at a stoichiometry of 2:2:2 with PWWP2A/B being mutually exclusive with the canonical NuRD subunits MBD2/3 and CHD3/4 (Figure 1A). Although the NuRD complex has been found to play a role in cellular differentiation and cell type commitment through regulation of pluripotency and cellular reprogramming (Luo et al., 2013; Mor et al., 2018; Reynolds et al., 2012), the role of the PWWP2-HDAC complex is still not well understood. Unlike the NuRD complex subunits MBD3 and CHD4, which are enriched at active enhancers and promoters (Bornelov et al., 2018; Shimbo et al., 2013), the PWWP2A variant NuRD complex is targeted to gene bodies. We previously showed that the PWWP2A localizes to gene bodies through binding to H3K36me3 via its C-terminal PWWP domain, where it regulates histone acetylation levels at highly transcribed genes (Zhang et al., 2018). Given that this novel HDAC complex was targeted to H3K36me3, we wondered whether the PWWP2-HDAC complex could fulfill the same function as the yeast Rpd3S HDAC complex in protecting against spurious transcription initiation. Cap analysis of gene expression (CAGE-seq) (Takahashi et al., 2012), a method widely applied to capture transcription initiation events (Andersson et al., 2014; Brocks et al., 2017; Fort et al., 2014), allowed us to precisely map and quantify all sites of transcription initiation at canonical promoters and spurious sites genome-wide in wild-type and *Pwwp2a/b* double knockout (DKO) mouse embryonic stem cells (mESCs). We used mESCs deleted for both PWWP2A and PWWP2B as our previous biochemical characterized showed these two paralogs can coexist in the same complex. Absence of PWWP2A/B in mESCs leads to increased levels of spurious transcription initiation particularly over the gene bodies of highly expressed genes. The profile of spurious initiation events upon PWWP2A/B depletion in mESCs contrasts with that in *Dnmt3b* knockout (KO) cells, suggesting that, although both gene body DNA methylation and histone deacetylation function downstream of H3K36me3 signaling, they have distinct roles in the suppression of spurious transcription initiation.

## Results

### Loss of PWWP2A/B in mESCs Results in Increase Spurious Transcription Initiation within Intragenic Sites

To investigate the role of the PWWP2A/B HDAC complex in spurious transcription initiation, we performed CAGE-seq in wild-type mESCs and two previously characterized *Pwwp2a/b* DKO mESC lines (clones A1 and C7) (Zhang et al., 2018). CAGE-seq using the nuclear RNA fraction as input was performed in triplicate for wild-type and *Pwwp2a/b* DKO C7 and in duplicate for DKO A1 (Figure 1A), and the biological replicates were merged owing to high reproducibility (Figure S1A). On average, each CAGE-seq library generated more than 20 million mapped reads (Table S1). The high sequencing depth allowed us to capture thousands of transcription initiation events (19134 CAGE peaks/clusters) in mESCs corresponding to annotated transcription initiation sites, spurious initiation sites, and transcription from enhancers. CAGE peaks were categorized as annotated promoter, intragenic (exonic and intronic), or intergenic TSSs (see Transparent Methods). Although the majority (∼73%) of the reads and the consensus CAGE peaks (∼63%) corresponded to GENCODE annotated promoter-TSSs (Table S1 and Figure 1B), we detected thousands of initiation events (37% of CAGE peaks) that mapped to exonic, intronic, and intergenic regions (Figure 1B) in the wild-type and DKO mESCs.

To distinguish between spurious transcription initiation events and enhancers (eRNAs), all CAGE peaks were compared with ATAC-seq and H3K27ac ChIP-seq peaks, which mark active promoters and enhancers. As expected, the majority of annotated promoter CAGE peaks overlapped with regions of high chromatin accessibility as assayed by ATAC-seq and H3K27ac (Figures 1C and S1B). Approximately half of intergenic CAGE peaks overlapped with ATAC-seq peaks suggesting that these transcripts may correspond to eRNA production at enhancers (Figure 1C) (Andersson et al., 2014). The majority of exonic and intronic CAGE peaks did not overlap with enhancers (ATAC-seq and H3K27ac peaks) (Figures 1C and S1B) suggesting that the majority of intragenic TSSs are spurious transcription initiation events and not eRNAs.

We next examined the underlying sequence features of annotated promoter-TSSs and non-annotated spurious TSSs detected in wild-type E14 cells. As expected, canonical annotated promoter-TSSs are enriched in common core promoter elements (GC box, CCAAT box) and TF motifs commonly found at promoters (ETS, YY1, NRF) (Figure 1D). Spurious TSSs are less enriched for fewer common core promoter elements but still contain promoter-related motifs (DCE, GC box, ETS) compared with background (Figure 1E). CAGE peaks corresponding to spurious TSSs also tend to be much narrower than peaks at annotated promoter-TSSs (Figure S1C). These results suggest that by CAGE the majority of detectable peaks come from canonical eukaryotic promoters, but thousands of spurious transcription initiation events still arise from weak cryptic promoters even in wild-type mESCs.

To determine the effect of PWWP2A/B deletion in mESCs, we intersected all CAGE peaks from wild-type E14 with those from the DKO clones. The vast majority of CAGE peaks in wild-type and *Pwwp2a/b* DKO mESCs show extensive overlap (Figures 1F, S1D, and S1E), suggesting that PWWP2A/B loss does not have a significant impact on the number of transcription initiation sites in the genome. We next performed differential expression analysis to determine whether PWWP2A/B deletion would upregulate transcription initiation from existing spurious sites. TSS peaks that increase in expression in *Pwwp2a/b* DKO cells compared with wild-type are identified as “Up,” and peaks that decrease are “Down,” and we observed many intragenic TSSs being upregulated or induced in mESCs lacking PWWP2A/B. At intragenic TSSs, the number of Up sites is 14.8 and 2.2 times more than the number of Down sites for exonic and intronic TSSs, respectively (Figures 1G and Table S2). In particular, exonic TSSs show increased levels of spurious transcription initiation upon PWWP2A/B loss compared with wild-type mESCs (Figures 1F–1H and S2). Annotated TSS and intergenic TSS peaks did not show a skewed sensitivity to PWWP2A/B loss, with similar numbers of peaks increasing or decreasing in the level of transcription initiation (Figure 1G).

### Spurious Sites Sensitive to PWWP2A/B Depletion Are Enriched at Intragenic Regions of Highly Expressed Genes

**Figure 2:**
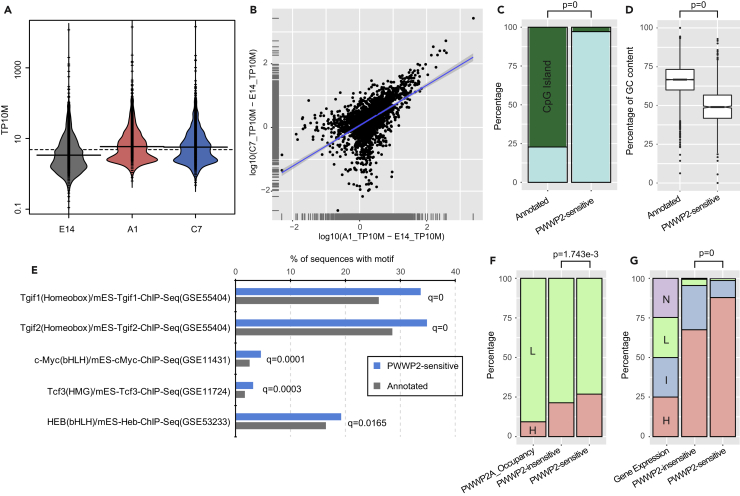
Intragenic Spurious Transcription Initiation Sites Arise Predominantly from Highly Expressed Genes (A) Transcription initiation expression value (TP10M) of all intragenic PWWP2-sensitive spurious initiation sites (Up events n = 2,146) in E14 and *Pwwp2a/b* DKO lines. See also Table S2. (B) Scatterplot shows the correlation of PWWP2-sensitive spurious TSSs detected in *Pwwp2a/b* DKO lines A1 and C7. See also Figure S3. (C) Percentage of annotated TSSs and PWWP2-sensitive spurious TSSs (n = 2,146) that intersect (dark green) or do not intersect (cyan) with CpG islands. The p value was calculated by a chi-square test using the frequency. (D) Boxplot of the %GC content for annotated TSSs (n = 11,043) and PWWP2-sensitive spurious TSSs (longer than 10 nt, n = 1,224). The p value was calculated using the two-sided Mann-Whitney test. (E) The top most enriched motifs in PWWP2-sensitive spurious TSS (blue bars) compared with annotated TSSs (gray bars), as detected by the Homer motif search analysis. (F) Percentage of spurious PWWP2-sensitive and PWWP2-insensitive TSSs occurring in genes with high (approximately top 10%) or low PWWP2A binding as defined by ChIP-seq (Zhang et al., 2018). (G) Percentage of spurious PWWP2-sensitive and PWWP2-insensitive TSSs arising from genes with High, Intermediate, Low, or No expression (obtained by sorting all genes by expression level in wild-type E14 mESCs and dividing into four equal groups). A majority of spurious events (both PWWP2-sensitive and insensitive) occur from highly expressed gene group, which a greater fraction of PWWP2-sensitive spurious TSS residing in the most highly expressed group. The p value was calculated by chi-square test using the frequency. See also Figure S4.

To further study the role of PWWP2A/B in regulating intragenic spurious initiation, we defined the subset of CAGE-seq peaks most sensitive to PWWP2A/B depletion. We removed intragenic TSS events that intersected with H3K27ac and ATAC-seq peaks, which may represent eRNAs, and classified the remaining 2,146 intragenic Up TSSs in both DKO clones as PWWP2-sensitive spurious TSSs (Figure 2A and Table S2). Owing to good correlation between the two clones A1 and C7 (Figures 2B and S3A), all downstream analysis was averaged for the two *Pwwp2a/b* DKO clones.

As discussed previously, motif analysis of the CAGE peaks for annotated and spurious TSSs in mESCs revealed that spurious TSSs are far less enriched for core promoter sequence motifs compared with annotated promoter-TSSs (Figures 1D and 1E). Furthermore, the majority (approximately 75%) of annotated promoter-TSSs overlapped with CpG islands and have high GC content (Figures 2C and 2D), whereas PWWP2-sensitive intragenic spurious transcription initiation peaks rarely overlap with CpG islands and are not GC-rich (Figures 2C and 2D). Lack of strong canonical promoter-like characteristics at spurious TSSs may explain the weaker transcription initiation capacity from these spurious intragenic cryptic promoter sites. Motif analysis comparing PWWP2-sensitive TSSs against annotated TSSs revealed that transcriptional repressors Tgif1/2 are significantly enriched at the flanking regions of PWWP2-sensitive spurious TSS in mESCs (Figure 2E) (Lee et al., 2015) and may suggest these cryptic promoters could be actively bound and repressed.

We next sought to characterize what type of genes were susceptible to PWWP2-sensitive spurious initiation. Gene ontology enrichment analysis revealed that genes harboring PWWP2-sensitive spurious TSSs are from a variety of biological pathways (Figure S4). Several RNA processing and surveillance pathways are also enriched, including RNA splicing, translation, and RNA nonsense-mediated decay (Figure S4). Previously we identified all genes bound by PWWP2A by ChIP-seq and classified them as being highly (9.4%) or lowly (90.6%) bound (Zhang et al., 2018). High-occupancy genes are more enriched for H3K36me3 than low-occupancy genes (Figure S3B). Here we see that all genes harboring intragenic spurious initiation sites are PWWP2A bound to some degree in mESCs, with a slightly greater proportion of PWWP2-sensitive spurious initiation events arising from high PWWP2A-occupied genes (Figure 2F). Strikingly, the greatest determinant correlated with the incidence of spurious transcription initiation is the level of gene expression, with the majority of spurious sites occurring within highly expressed genes (Figure 2G). Specifically, 88% of PWWP2A-sensitive spurious TSSs occur in the top 25% of genes ranked by expression (n = 1,108, see Transparent Methods) (Figure 2G). Together, these results indicate that PWWP2-sensitive spurious TSSs lack the underlying sequence features of canonical annotated promoters such as enrichment of many core promoter motifs, high GC and CpG island content, but contain weak promoter elements that are susceptible to spurious initiation events especially within highly expressed genes.

### Loss of PWWP2A/B and DNMT3B Have Distinct Effects on Spurious Transcription Initiation

**Figure 3:**
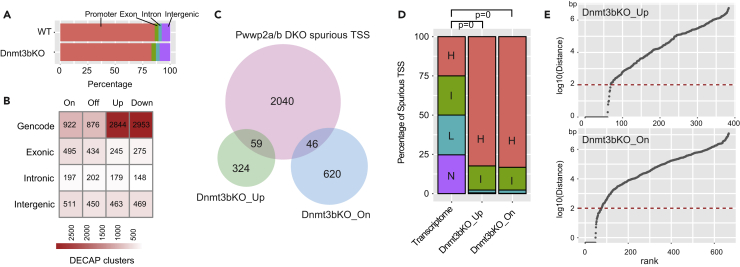
PWWP2A/B and DNMT3B Loss Have Different Effects on Spurious Transcription Initiation (A) The percentage of consensus TSSs detected by DECAP-seq in wild-type and *Dnmt3b* KO mESCs from GSE72854 categorized as annotated promoter, exonic, intronic, intergenic TSSs. (B) TSS peaks for wild-type and *Dnmt3b* KO cells categorized as Gencode annotated promoters, exonic, intronic, and intergenic TSSs and divided into four groups based on behavior: On (only present in *Dnmt3b* KO), Off (only present in wild-type), Up (upregulated in KO), and Down (downregulated in KO). See also Tables S1 and S3 and Figure S5. (C) Overlap between PWWP2-sensitive spurious TSSs (n = 2,146) and DNMT3B-sensitive spurious TSSs (On n = 666 and Up = 383). (D) Percentage of DNMT3B-sensitive spurious TSSs arising from genes with High, Intermediate, Low, or No expression (obtained by sorting all genes by expression level in wild-type E14 mESCs and dividing into four equal groups). The p values were calculated by chi-square test using the frequency. (E) Distribution of the distance in base pairs of the closest PWWP2-sensitive spurious TSS relative to every DNMT3B-sensitive TSSs (Up and On). Dashed line indicates presence of a PWWP2-sensitive TSS within 100 bp.

The DNA methyltransferase DNMT3B, like PWWP2A/B, is also selectively recruited to gene bodies through binding to H3K36me3 via its PWWP domain (Rondelet et al., 2016). A prior study found that loss of DNMT3B in mESCs leads to intragenic DNA hypomethylation and increased spurious transcription initiation events (Neri et al., 2017). Given that both of these pathways are downstream of H3K36me3 and contribute to regulating the epigenetic landscape at gene bodies, we sought to determine their relationship with one another. We applied our pipeline to analyze previously published data from *Dnmt3b* KO mESCs, which was performed using DECAP-seq, an alternative to CAGE-seq for profiling sites of transcription initiation. Consistent with the published study, we found that most DECAP TSSs correspond to Gencode annotated promoters (Figure 3A). We classified all DNMT3B TSSs following the terminology used in the previous DNMT3B study—TSSs absent in wild-type but present in *Dnmt3b* KO are categorized as “On,” TSSs present in wild-type cells but lost in *Dnmt3b* KO are “Off,” upregulated TSSs in *Dnmt3b* KO are “Up,” and downregulated TSSs in *Dnmt3b* KO are “Down” (Figures 3B and Table S3). Unlike in *Pwwp2a/b* DKO mESCs, where sites of spurious initiation almost perfectly overlap with wild-type but show different levels of transcription initiation, either increased (Up) or decreased (Down) (Figures 1G, S1D, and S1E), *Dnmt3b* KO mESCs showed many On and Off sites indicating that loss of DNMT3B-mediated DNA methylation led to a redistribution of spurious sites in mESCs (Figure 3B). Another distinction between the two pathways was that, *Pwwp2a/b* deletion led to preferential upregulation of intragenic spurious TSSs, whereas *Dnmt3b* deletion resulted in an equivalent number of Up and Down spurious sites.

We next identified DNMT3B-sensitive spurious TSSs as all intragenic On and Up TSSs after removal of sites that overlapped with ATAC-seq and H3K27ac peaks. The overlap between sites (genomic positions) of PWWP2-sensitive and DNMT3B-sensitive spurious initiation is very low (Figure 3C). However, like PWWP2-sensitive sites, DNMT3B-sensitive spurious initiation also occurs predominantly at highly expressed genes (Figure 3D) and displays similar promoter width as well as CpG Island and GC content as PWWP2-sensitive spurious TSSs (Figures 2C, 2D, and S5A–S5C). Despite both DNMT3B-sensitive and PWWP2-sensitive spurious residing within highly expressed genes, there is little overlap between sites (Figure 3C) with the vast majority of DNMT3B-sensitive spurious TSS more than 100 bp away from the closest neighboring PWWP2-sensitive spurious TSS (Figure 3E).

### Transcription and Chromatin Signatures Underlying Spurious Transcription Initiation

**Figure 4:**
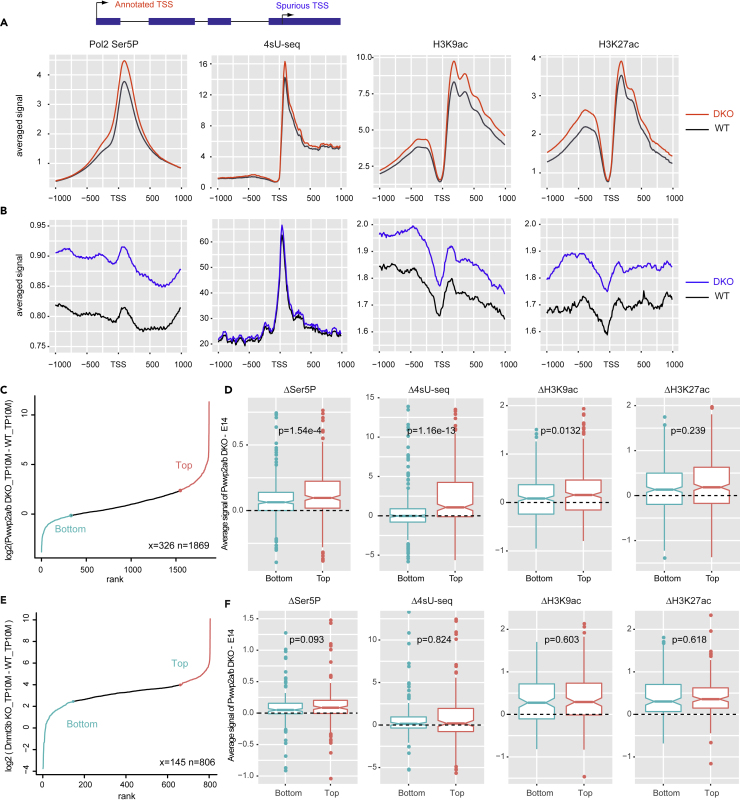
Elevated RNAPII and Histone Acetylation at Spurious Transcription Initiation Sites in *Pwwp2a/b* DKO mESCs (A) Metagene profiles of RNA Pol II Ser5P, 4sU-nascent transcription, H3K9ac, and H3K27ac at annotated TSSs ± 1 kb (n = 8,164) of wild-type and *Pwwp2a/b* DKO mESCs. (B) Metagene profiles RNA Pol II Ser5P, 4sU-nascent transcription, H3K9ac, and H3K27ac at the PWWP2-sensitive spurious TSSs ± 1 kb. Only TSSs that are 2 kb away from each other were considered (n = 1,869). (C) PWWP2-sensitive spurious TSSs ranked by the level of upregulation in *Pwwp2a/b* DKO compared with wild-type. Only sites 2 kb away from each other were considered (n = 1,869). Bottom group: spurious sites that show small increase in CAGE expression upon PWWP2A/B loss (cyan). Top group: spurious sites that show large increase in CAGE expression upon PWWP2A/B loss (red). (Top and Bottom group: x = 326). (D) Boxplots of the distribution of relative differences of the Top and Bottom PWWP2-sensitive spurious sites with respect to the level of Pol II Ser5P, nascent transcription, H3K9ac and H3K27ac. p Values were calculated and using the two-sided Mann-Whitney test between *Pwwp2a/B* DKO and wild-type E14 mESCs. (E) DNMT3B-sensitive spurious TSSs ranked by the level of upregulation in *Dnmt3b* KO compared with wild-type. Only TSSs 2 kb away from annotated TSS and 100 bp away from PWWP2-sensitive TSSs were considered (n = 806). Red and cyan indicate top (most sensitive) and bottom group (least sensitive) sites, respectively (x = 145, the same proportion as in C). (F) Boxplots of the distribution of relative differences between *Pwwp2a/b* DKO and E14 for Top and Bottom groups DNMT3B-spurious sites from (E) with respect to the level of Pol II Ser5P, nascent transcription, H3K9ac and H3K27ac. p Values were calculated and using the two-sided Mann-Whitney test *Pwwp2a/B* DKO and wild-type E14 mESCs. See also Figure S6.

We next explored the dynamics of Pol II binding, nascent transcription, and histone acetylation at PWWP2-sensitive and DNMT3B-sensitive spurious transcription initiation sites. Canonical transcription initiation sites are enriched for initiated RNA Polymerase II phosphorylated at serine 5 (RNA Pol II Ser5P) surrounding the TSS. Pol II Ser5P binding thus serves as an orthogonal approach to examine sites of spurious transcription initiation (Phatnani and Greenleaf, 2006). To reduce noise from neighboring peaks, only single peaks in a 2-kb window were considered for downstream analysis for both the annotated TSSs and spurious TSSs. By comparing Pol II Ser5P ChIP-seq in E14 and *Pwwp2a/b* DKO cells, we found that Pol II Ser5P signal slightly peaks just downstream of the spurious TSSs resembling annotated promoter-TSSs (Figure 4A), although at a much lower levels (Figures 4A and 4B). Pol II Ser5P levels are also increased in *Pwwp2a/b* DKO when compared with E14 cells (Figure 4B). These intragenic spurious initiation sites also showed a local increase of nascent RNA transcription compared with background as detected by 4sU nascent RNA-seq in wild-type E14 and *Pwwp2a/b* DKO lines (Figure 4B). In contrast to annotated TSSs, which display a strong asymmetric pattern around the peak indicative of a bias toward the sense direction and weak antisense transcription, the nascent RNA transcription around spurious transcription initiation sites is more balanced around the peak suggesting that the transcription driven by spurious TSSs is weakly expressed bidirectionally or may not produce long RNA products (Figure 4B).

Acetylation of H3K27 and H3K9 is enriched at active genes, peaking around the TSS. Previously we showed that loss of PWWP2A/B HDAC complex leads to increased histone acetylation at active gene promoters and gene bodies (Zhang et al., 2018). We assessed the pattern of H3K27ac and H3K9ac at PWWP2-sensitive spurious TSSs defined in this study and found the signature resembles that of annotated TSSs, with a trough at the spurious TSS and peaks at the +1 and −1 nucleosome positions (Figures 4A and 4B). Spurious TSSs show increased acetylation levels in *Pwwp2a/b* DKO cells compared with wild-type mESCs (Figures 4B and S6B). We calculated the difference in RNA Pol II Ser5P, 4SU-seq, H3K9ac, and H3K27ac levels between *Pwwp2a/b* DKO and wild-type E14 and found all of them to be enriched around the spurious TSSs in the DKO (Figures S6A and S6B).

We divided the PWWP2-sensitive spurious TSSs sites into two groups based on the degree of increase in spurious transcription initiation compared with wild-type, “Top” (large increase in DKO compared with E14) and “Bottom” (small increase in DKO compared with E14), using TP10M of each CAGE peak (Figure 4C). There are significantly higher levels of initiating Pol II Ser5P, nascent RNA transcription, and H3K9ac at Top sites compared with Bottom sites (Figure 4D). This suggests that increased histone acetylation upon PWWP2A/B loss correlates with increased initiation and transcription from spurious sites, and the degree of change correlates with sensitivity to PWWP2A/B loss. In parallel we performed the same analysis for DNMT3B-sensitive spurious TSSs and found that spurious sites more (Top) or less (Bottom) sensitive to DNMT3B loss did not show differences in the level of initiating Pol II, nascent RNA production, or histone acetylation (Figures 4E, 4F, S6C, and S6D). These features, which explain sensitivity to PWWP2A/B loss, do not explain differences between spurious sites arising from DNMT3B depletion.

In summary, our analysis of the chromatin environment at spurious TSSs show that these spurious initiation sites show increased recruitment of initiating Pol II Ser5P, elevated levels of nascent transcription, and histone acetylation relative to the surrounding background. Additionally, upon PWWP2A/B loss, increased levels of spurious initiation are correlated with elevated histone acetylation and increased engaged RNA Pol II Ser5P and nascent transcription compared with wild-type. Although PWWP2A/B appears to regulate spurious transcription initiation at the level of histone acetylation and recruitment of initiating Pol II Ser5P, the DNMT3B pathway may regulate spurious initiation through altering binding of DNA-methylation sensitive factors (as discussed in Neri et al., 2017).

## Discussion

Transcription initiation is a highly coordinated process that establishes accurate patterns of gene expression and ensures the production of functional transcripts. Here we describe how cross talk between two epigenetic pathways H3K36me3 and histone acetylation suppresses spurious transcription initiation from the gene bodies of active genes. H3K36me3 is deposited co-transcriptionally with the elongating RNA Pol II and exclusively marks the gene bodies of actively transcribed genes (Kizer et al., 2005). Acetylation of H3K9 and H3K27 is enriched at active promoter regions and regulates RNA Pol II initiation and pause release (Gates et al., 2017; Stasevich et al., 2014). Here we describe a pathway in metazoans, in which H3K36me3 recruitment of the PWWP2A/B HDAC complex represses spurious transcription initiation from the gene bodies of actively transcribed genes. This prevents RNA Pol II from initiating at cryptic promoters within coding regions and the production of aberrant transcripts, which could be harmful to cellular homeostasis.

PWWP2A/B binds H3K36me3 through a C-terminal PWWP domain, a module present in many other H3K36me3-binding proteins (Qin and Min, 2014; Vermeulen et al., 2010; Vezzoli et al., 2010; Wen et al., 2014). Previously we and others showed that PWWP2A/B forms a stable complex HDAC1/2 and MTA1/2/3 (Link et al., 2018; Zhang et al., 2018). In this study, we find that loss of PWWP2A/B in mESCs leads to increases in the level of spurious transcription initiation, especially over intragenic gene body regions.

Similar to studies in *S. cerevisiae*, we find that, although spurious transcripts are lowly expressed, they are highly prevalent in number within gene bodies in mESCs (Lu and Lin, 2019; Neil et al., 2009). Spurious initiation sites contain some core promoter elements like the GC box, DCE, and ETS motifs but are far less enriched for these motifs than canonical eukaryotic promoters (Haberle and Stark, 2018; Hollenhorst et al., 2004; Lee et al., 2005; Roy and Singer, 2015). Additionally, spurious initiation sites also lack the high GC and CpG island content associated with eukaryotic promoters (Deaton and Bird, 2011; Haberle and Stark, 2018; Roy and Singer, 2015). The vast majority of spurious transcription initiate from highly expressed genes, and these sites are marked by initiating Pol II Ser5P, which increases further upon PWWP2A/B loss. The increased level of spurious transcripts at highly expressed genes may reflect increased levels of hyperacetylation, increased chromatin accessibility and nucleosome turnover, and a high local concentration of coactivators and transcriptional machinery, which together lead to binding and initiation of RNA Pol II within intragenic sites. Our data support the model where promiscuous initiation by Pol II occurs more frequently at highly expressed genes and that spurious transcription initiation is an inevitable by-product of transcription (Wade and Grainger, 2018). Multiple mechanisms besides the H3K36me3-HDAC pathway have been found to play a role in countering spurious initiation and the production of spurious transcripts (Gouot et al., 2018; Neri et al., 2017; Scandaglia et al., 2017), with intragenic DNA methylation by DNMT3B as another pathway downstream of H3K36me3 that suppresses spurious transcription initiation.

We find that loss of the PWWP2A/B HDAC complex has a different profile than loss of DNMT3B, and PWWP2-sensitive and DNMT3B-sensitive spurious TSSs are largely non-overlapping. The only features common to both pathways was that intragenic spurious transcription initiate predominantly from the highly expressed genes. Our data suggest that PWWP2A/B suppresses transcription initiation through regulating histone acetylation and Pol II initiation, whereas DNA methylation may regulate the binding of methylation-sensitive TFs to suppress initiation at intragenic sites. Although PWWP2A/B loss leads to increased levels of initiation from pre-existing intragenic sites found in mESCs, loss of DNMT3B leads to redistribution of sites from which spurious transcripts initiate. It appears that in mammalian organisms these two epigenetic mechanisms cooperate downstream of H3K36me3 to control transcription fidelity by ensuring that initiation occurs predominantly at strong promoter regions and is actively suppressed from intragenic regions.

### Limitations of the Study

There are two points that are outside the scope of this study and will require more experimental work to fully elucidate, these being whether PWWP2A and PWWP2B have redundant functions and the role of the PWWP2A/B-HDAC complex at intergenic regions.

1. Overlapping and paralog-specific differences of PWWP2A and PWWP2B

In this work, we used mESCs where both the Pwwp2a and Pwwp2b genes have been knocked out. Our justification for using the double knockout comes from biochemical characterization of the complex in our previous work (Zhang et al., 2018), which shows that two copies of PWWP2A bind to the MTA1-dimer (stoichiometry of 2:2), PWWP2A and PWWP2B can coexist in the same complex, and the PWWP domains have the same affinity for H3K36me3. However, despite high sequence conservation, there are differences in the domain composition of the two paralogs (see Figure S7) with PWWP2B lacking the N-terminal proline-rich region present in PWWP2A. Both paralogs are expressed in mESCs but could have differences in tissue-specific expression and functions. Further work is needed to address whether there are paralog-specific differences in this family of proteins.

2. Intergenic regions that show sensitivity to PWWP2A/B loss

Our characterization of spurious transcription initiation focused predominantly on intragenic spurious TSSs, which were sensitive to PWWP2A/B deletion. Although intragenic spurious TSSs were much more sensitive to loss of PWWP2A/B, some intergenic spurious TSSs were also induced. These intergenic sites are not enriched for H3K36me3, so we cannot subscribe these events to H3K36me3-mediated recruitment of PWWP2A/B. Aside from the H3K36me3-binding PWWP domain, there is a H2A.Z-binding domain, which could recruit PWWP2A/B to chromatin, but whether this could explain intergenic spurious TSSs events remains to be examined.

### Resource Availability

#### Lead Contact

Further information and requests for resources and reagents should be directed to and will be fulfilled by the corresponding author, Tianyi Zhang (tzhang17@ic.ac.uk).

#### Materials Availability

Materials and protocols used in this study are available from the authors upon request. This study did not generate new unique reagents.

#### Data and Code Availability

CAGE-seq and processed bigwig files generated in this study have been deposited in Gene Expression Omnibus (GEO) under GSE148382. Code used in this study has been deposited in github, https://github.com/guifengwei/PWWP2-CAGE-seq.

## Methods

All methods can be found in the accompanying Transparent Methods supplemental file.

## Supplemental Information

Document S1. Transparent Methods and Figures S1–S7

Table S1. Summary of CAGE-Seq Mapped Reads, Related to Figures 1 and 3

Table S2. Annotated and Spurious CAGE-Seq Peaks in E14 and *Pwwp2a/b* DKO mESCs, Related to Figure 2

Table S3. Annotated and Spurious DECAP-Seq Peaks in *Dnmt3b* KO mESCs, Related to Figure 3
