# Supplementary material for: The PWWP2A Histone Deacetylase Complex Represses Intragenic Spurious Transcription Initiation in mESCs
Source: iScience. 2020 Oct 29;23(11):101741. doi: 10.1016/j.isci.2020.101741 (PMC7670215; doi:10.1016/j.isci.2020.101741)
Supplement: Document S1. Transparent Methods and Figures S1–S7 [file mmc1.pdf]

iScience, Volume *23*

Supplemental Information

The PWWP2A Histone Deacetylase Complex Represses Intragenic Spurious Transcription Initiation in mESCs

Guifeng Wei, Neil Brockdorff, and Tianyi Zhang

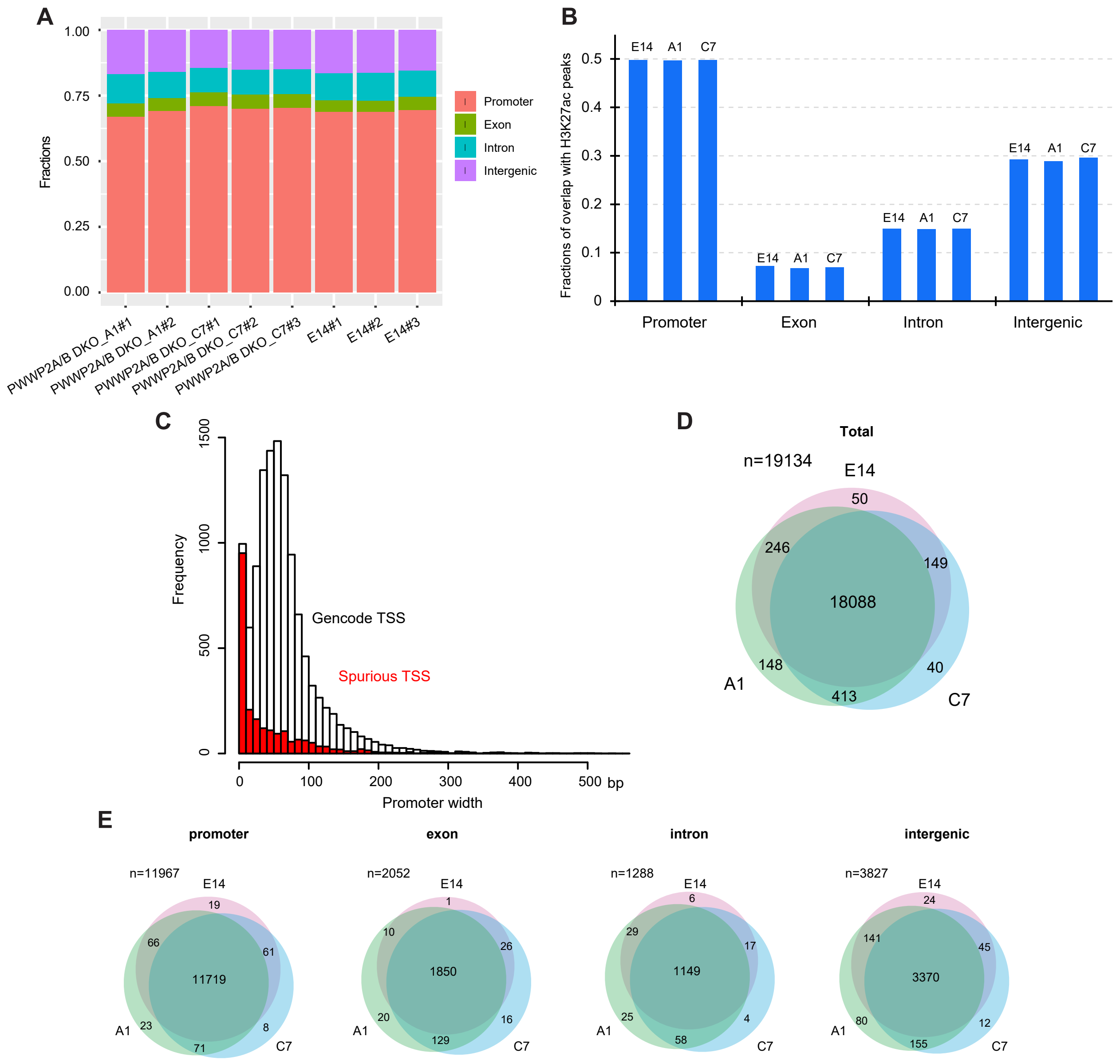

Figure S1. Annotation of CAGE-seq reads to GENCODE genomic features, related to Figure 1.

(A) Fraction of CAGE-seq reads in promoter, exon, intron, and intergenic regions in E14 wildtype and in *Pwwp2a/b* DKO mESC lines A1 and C7. Also see Table S1.

(B) The fraction of consensus TSS from all the categories that overlap with H3K27ac-seq peaks in mESC for E14 and *Pwwp2a/b* DKO lines.

(C) Histogram shows the width distribution for Gencode annotated TSSs (n=11967) and Spurious TSSs (n=2146) respectively. See also Table S2.

(D-E) Venn diagrams show the overlap of CAGE peaks (TP10M>1) between wildtype E14 and *Pwwp2a/b* DKO lines. All CAGE peaks are shown in (D) and peaks present in promoter, exonic, intronic, and intergenic sequences are shown separately in (E).

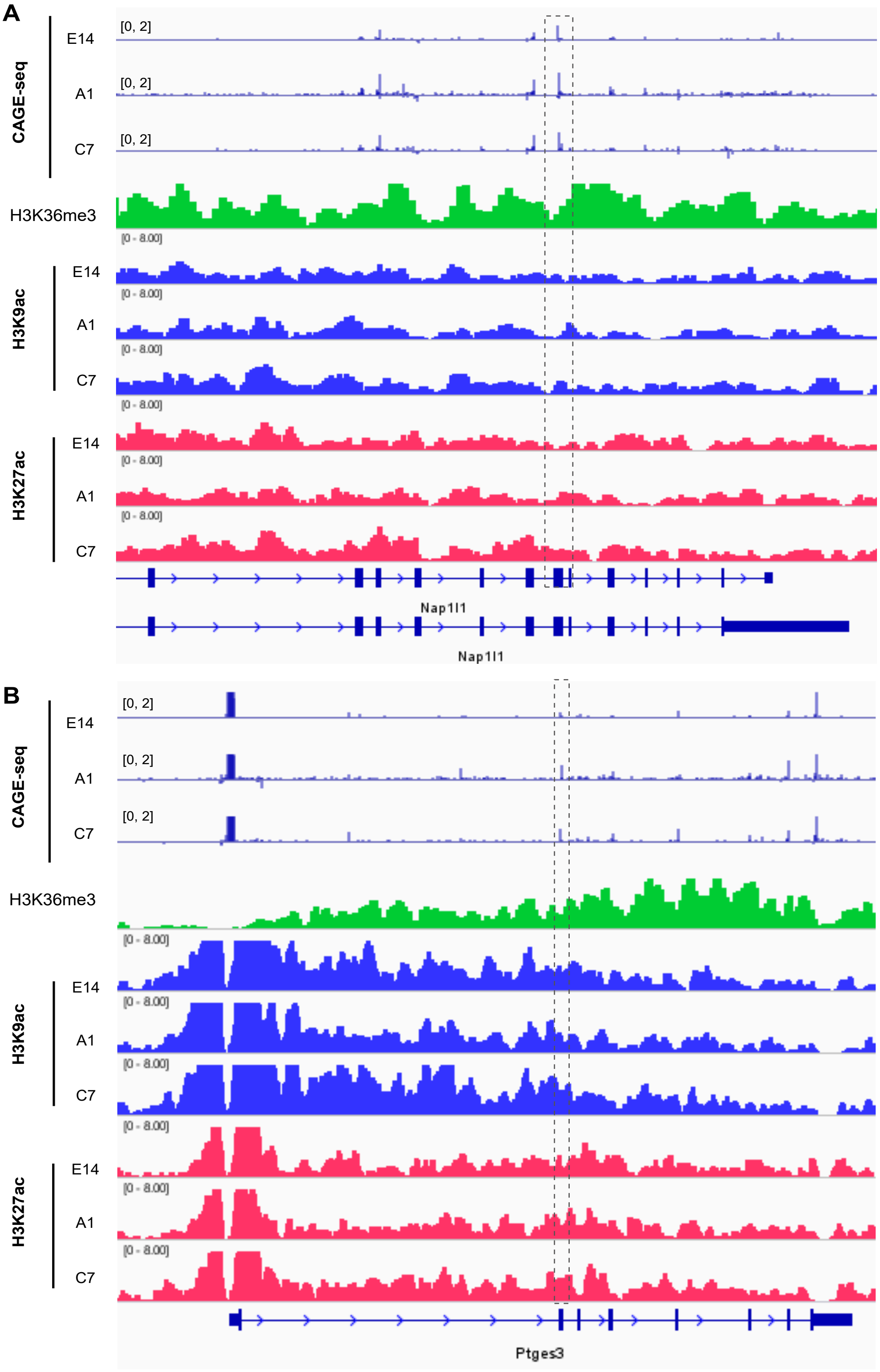

Figure S2. Chromatin landscape at representative PWWP2-sensitive spurious TSSs, related to Figure 1.

(A-B) IGV browser view of the profiles for CAGE, H3K36me3, H3K9ac, and H3K27ac data in E14 (WT) cells and *Pwwp2/b* DKO cells. Dashed box indicates the representative intragenic spurious TSS at (A) *Nap1l1* locus and (B) *Ptges3* locus. Same type of data is adjusted to same scale.

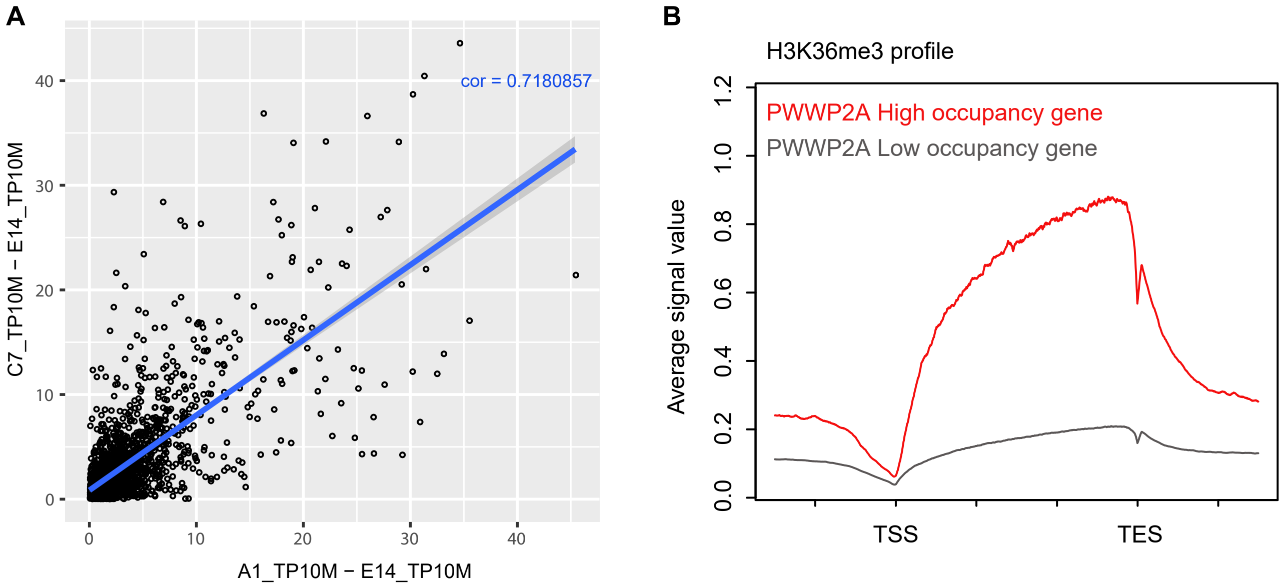

Figure S3. Correlation of CAGE-seq in between *Pwwp2a/b* lines, related to Figure 2.

(A) Correlation of the PWWP2-sensitve intragenic spurious TSS between *Pwwp2a/b* DKO clones A1 and C7 (excluding the extreme outliers) with the correlation score at top right. Pearson correlation was calculated based on non-log-transformed values. The dashed blue line indicates the linear regression. (B) Metaprofile of H3K36me3 level at high (red) and low (grey) PWWP2A occupancy genes as defined by ChIP-seq (Zhang et al. 2018).

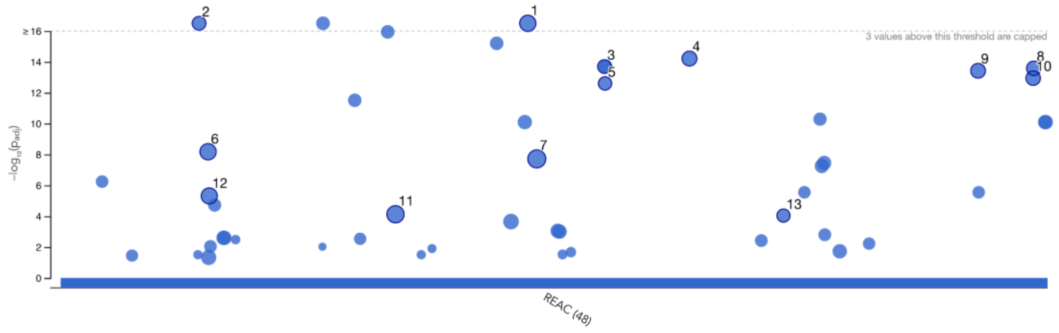

ID	Source	Term ID	Term Name	p_{adj} (query_1)
1	REAC	REAC:R-MMU-8953854	Metabolism of RNA	2.264×10^{-34}
2	REAC	REAC:R-MMU-72737	Cap-dependent Translation Initiation	2.563×10^{-17}
3	REAC	REAC:R-MMU-975957	Nonsense Mediated Decay (NMD) enhanced by the Exon Junction Complex (EJC)	2.003×10^{-14}
4	REAC	REAC:R-MMU-72203	Processing of Capped Intron-Containing Pre-mRNA	6.092×10^{-15}
5	REAC	REAC:R-MMU-975956	Nonsense Mediated Decay (NMD) independent of the Exon Junction Complex (EJC)	2.490×10^{-13}
6	REAC	REAC:R-MMU-1640170	Cell Cycle	6.640×10^{-9}
7	REAC	REAC:R-MMU-392499	Metabolism of proteins	1.924×10^{-8}
8	REAC	REAC:R-MMU-72163	mRNA Splicing - Major Pathway	2.592×10^{-14}
9	REAC	REAC:R-MMU-72766	Translation	3.779×10^{-14}
10	REAC	REAC:R-MMU-72172	mRNA Splicing	1.135×10^{-13}
11	REAC	REAC:R-MMU-74160	Gene expression (Transcription)	7.462×10^{-5}
12	REAC	REAC:R-MMU-69278	Cell Cycle, Mitotic	4.826×10^{-6}
13	REAC	REAC:R-MMU-450531	Regulation of mRNA stability by proteins that bind AU-rich elements	9.060×10^{-5}

Figure S4. Biological pathways enrichment analysis, related to Figure 2.

Genes harbouring spurious transcription initiations were used for Gene Ontology enrichment analysis with online tools g:Profiler. Representative biological pathways and adjust p-values from Reactome are shown.

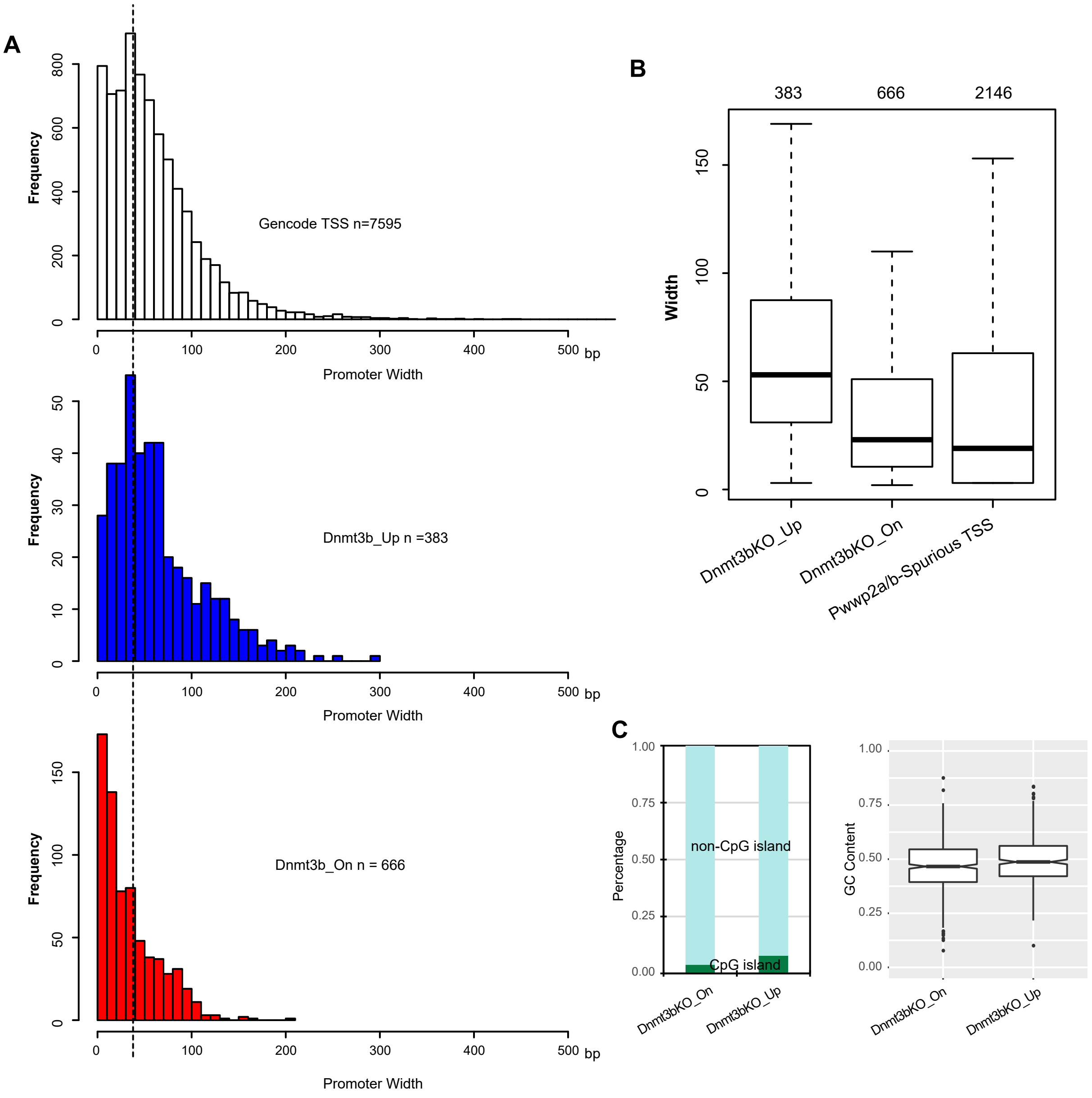

Figure S5. Width distribution and sequence content of DNMT3B-sensitive spurious TSSs, related to Figure 3.

(A) Histograms show width of consensus TSS called from DECAP-seq by CAGEr pipeline. Top panel – Gencode annotated TSSs, middle panel – Dnmt3bKO UP TSSs, and bottom panel – Dnmt3bKO On TSS. TSS numbers are indicated and the dashed line shows the median width in Gencode TSSs. See also Table S3.

(B) Boxplot shows the TSS width comparison in Dnmt3bKO_Up, Dnmt3bKO_On, and PWWP2-sensitive Spurious TSSs. TSS numbers are shown on the top. Boxes indicate the median and IQRs, with whiskers indicating 1.5× the IQR, outliners are not shown.

(C) Percentage of Dnmt3bKO_ On and Up TSSs that lie within a CpG island (Left), and their GC content (Right).

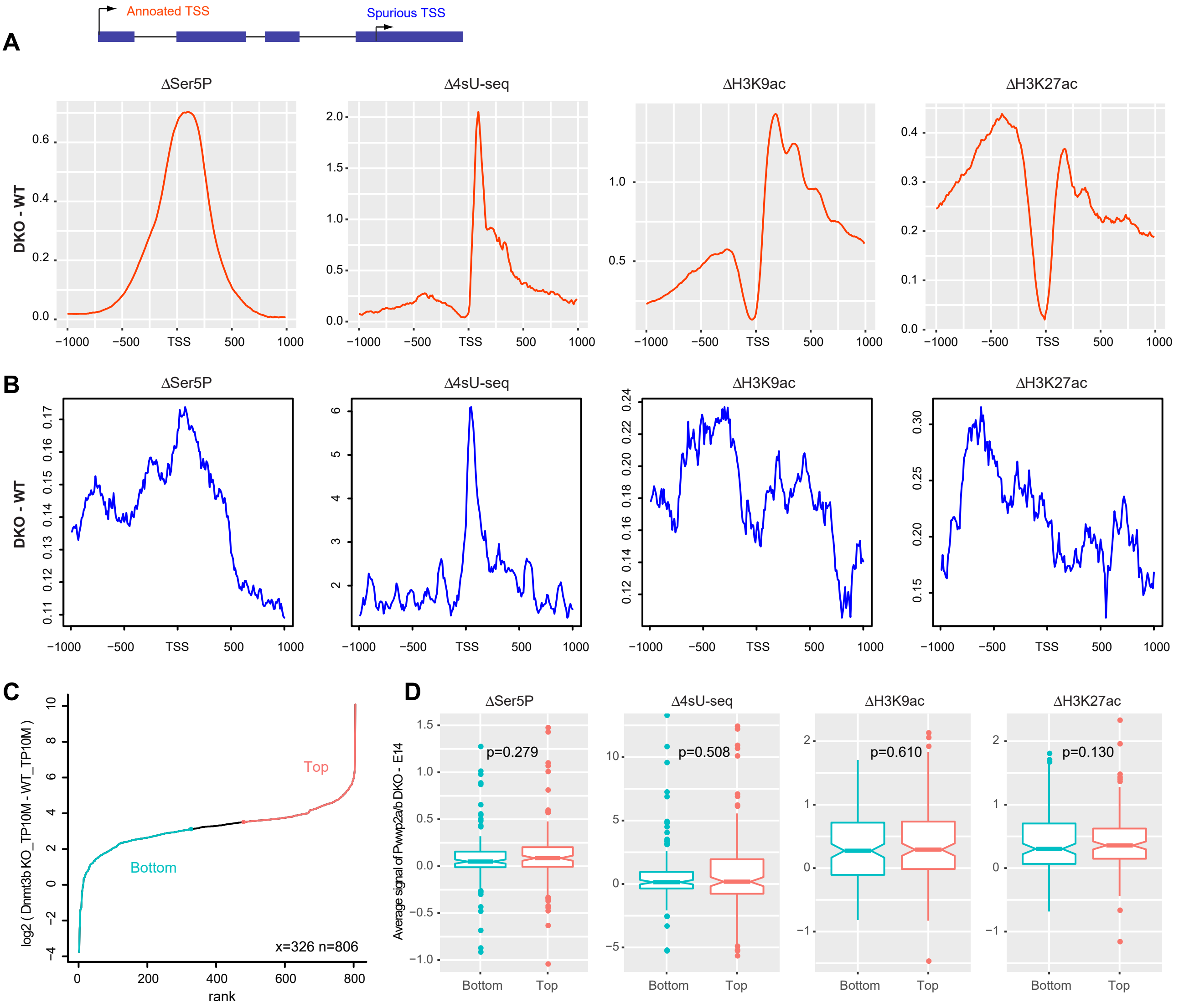

Figure S6. Changes of chromatin signatures and nascent transcription around PWWP2-sensitive spurious TSSs or DNMT3B sensitive spurious TSSs, related to Figure 4.

(A-B) Metaprofile of Pol II Ser5P, 4sU, H3K9ac, and H3K27ac of *Pwwp2a/b* DKO - E14 signal at the 1000 bp flanking annotated TSS (red) (A) and spurious TSSs (blue) (B), which all show a signal > 0 indicating greater occupancy in *Pwwp2a/b* DKO compared to E14.

(C-D) Same as Figure 4E,F but use the top and bottom 326 Dnmt3bKO_On and _Up spurious TSSs, matching the number in Figure 4C,D. In Figure 4C,D and 4E,F, the proportion is matched.

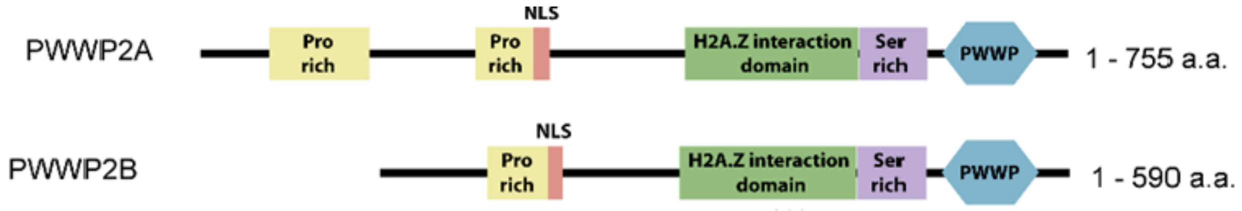

Figure S7. Domain composition of PWWP2A and PWWP2B, related to Figure 1 and Limitations of this study.

TRANSPARENT METHODS

Cell culture

Wildtype E14 and *Pwwp2a/b* DKO mouse embryonic stem cells were grown in Dulbecco's Modified Eagle Medium (DMEM, from Life Technologies) supplemented with 10% foetal calf serum (Seralab), 2 mM L-glutamine, 1X non-essential amino acids, 50 µM *β*-mercaptoethanol, 50 g/mL penicillin/streptomycin (all from invitrogen) and 1000 U/mL of LIF in tissue culture dishes coated with PBS+1% gelatine. Cells were passaged using 0.05% trypsin-EDTA (Life Technologies) with 2% Chicken Serum (Life Technologies) and frozen in FCS +10% DMSO.

Nuclear RNA extraction

For the extraction of nuclear RNA, cells were lysed for 5 minutes on ice in lysis buffer (20 mM Tris pH 7.5, 150 mM NaCl, 5% glycerol, 0.5% NP-40, 1x protease inhibitor) then centrifuged at 2000 rpm to pellet nuclei. The nuclei were washed once in cold PBS then directly lysed in Trizol followed by standard RNA extraction. Nuclear RNA was prepared in biological replicates for wildtype and *Pwwp2a/b* DKO lines as the input RNA for CAGE-seq.

CAGE-seq library construction

Libraries for all samples (3 replicates for wildtype E14, 3 replicates for *Pwwp2a/b* DKO clone C7, and 2 replicates for DKO clone A1) were prepared using the nAnT-iCAGE-seq kit purchased from cage-seq.com. Briefly, 5 µg of nuclear RNA for each sample was primed (random primers) and reverse transcribed to cDNA, followed by biotinylation of and capture of the 7-methylguanosine cap of all RNA Polymerase II transcripts. Adapters containing 3bp barcodes were ligated and samples were purified and multiplexed. As the nAnT-iCAGE-seq protocol involves no PCR amplification step, the multiplexed pool was quantified by HS DNA qubit denatured and loaded into the Illumina NextSeq 500 for single end 80bp sequencing.

CAGE-seq data analysis

Barcodes were extracted from pooled single-end RNA-seq reads and moved to the header line. The resulting fastq files were demultiplexed using the 3-letter barcodes, and then mapped to mm10 genome by STAR (v2.4.2a) (Dobin et al., 2013) with default parameters expect (*--outWigType bedGraph read1_5p --outFilterMultimapNmax 1 --outFilterMismatchNmax 4 --seedSearchStartLmax 15 --alignEndsType EndToEnd*). Only uniquely mapped reads were kept for further analysis. The

reads mapping summary for each sample are listed in Supplementary Table 1. CAGEr (1.28.0) (Haberle et al., 2015) was employed for CAGE-seq data analysis, including TSS detection, clustering, aggregation, promoter width (qLow=0.1 and qUp=0.9), and quantification (See attached codes). The CAGE-seq libraries were normalized (powerLaw) and TP10M (tags per million) scores were calculated as 10 million mapped reads in this study. TSS clusters shorter than 20bp from same strand were further aggregated to consensus peaks across replicates. GENCODE vM22 (comprehensive annotation) were used to annotate all CAGE peaks into four groups: promoter (Gencode), exonic, intronic, and intergenic. CAGE peaks were also compared for overlap with ATAC-seq and H3K27ac (high at promoters and enhancers). Differential expression analysis comparing CAGE expression in *Pwwp2a/b* DKO vs wildtype was performed for all TSSs, and classified as upregulated or downregulated (Brocks et al., 2017). To obtain the list of PWWP2-sensitive TSSs (n=2146), exonic and intronic sites were combined and sites potentially corresponding to eRNAs (sites which overlap with ATAC and H3K27ac peaks) were removed. Intragenic spurious sites which are not consistently upregulated in *Pwwp2a/b* DKO were regarded as PWWP2-insensitive spurious TSS (n=1108). The CAGE-seq overlap among samples in different groups (Figure S1D,E) were performed by comparing TSSs with TP10M>=1. Gene expression group were calculated from wildtype E14 RNA-seq and gene sets with high or low PWWP2A occupancy were obtained from previous ChIP-seq study (Zhang et al., 2018). CpG island annotation was retrieved from mm10 UCSC genome browser. The detailed scripts used for CAGE-seq analysis are available.

CAGE-seq mapped reads were split into positive and negative strands according to the flag filed in BAM file with Samtools (1.3) (Li et al., 2009), and then visualized in UCSC or IGV genome browser. Gene Ontology enrichment analysis of genes harbouring spurious TSSs from *Pwwp2a/b* DKO cells were performed using online tools g:Profiler (Raudvere et al., 2019). Bedtools (2.25.0) (Quinlan and Hall, 2010) were used to annotate TSSs detected from CAGE-seq. Annotated TSSs and PWWP2-sensitive spurious TSSs that are 2kb away from annotated TSS were taken for metagene profile, and DANPOS2 (Chen et al., 2013) was used for metagene profile with parameters setting (*--flank_up 1000 --flank_dn 1000 --heatmap 1 --bin_size 10 --excludeP 0.005*). Homer software (Heinz et al., 2010) was used to calculate motif enrichment. For discovery of motifs enriched in spurious TSS or annotated TSS compared to background, the 200bp (100nt upstream + 100nt downstream) region flanking spurious or annotated TSS were extracted as input, and random 200bp genomic sequence matched for GC content were served as background (*findMotifs.pl promoter.fa mouse output_dir -fasta random.fa*). For discovery of motifs enriched in spurious TSS over annotated TSSs, 200bp regions flanking spurious TSSs were used as input and 200bp regions flanking annotated TSSs were used as background (*findMotifs.pl spurious.promoter.fa mouse*

output_dir -fasta annotated.promoter.fa). R (3.6) package ggplot2 (3.2.1), dplyr (0.8.4), pheatmap (1.0.12), and beanplot (1.2) were used for statistics analysis and plot.

Reanalysis of *Dnmt3b* KO DECAP-seq and comparison with *Pwwp2/b* DKO data

The same CAGEr and gene expression group categorisation pipeline was applied to the DECAP-seq data generated from wildtype and *Dnmt3b* KO mESCs in the Neri et al. study, retrieved from GSE72854 (Neri et al., 2017). DECAP peaks were categorised as annotated promoter-TSS, exonic, intronic, or intergenic. DECAP peaks detected in wildtype cells were overlapped with DECAP peaks in *Dnmt3b* KO cells and sorted into four states as described in the Neri et al. study – "On": peaks present only in KO, "Off": peaks present only in wildtype, Up: upregulated in KO, and Down: downregulated in KO. Dnmt3bKO_Up and _On TSSs which overlapped neither with ATAC nor H3K27ac peaks were merged for meta-analysis, and only TSSs which are 2kb away from annotated TSSs and 100bp away from PWWP2-sensitive TSSs were taken for analysis. Intersection and distance between DNMT3B-sensitive and PWWP2-sensitive spurious TSSs were calculated using Bedtools (2.25.0) (Quinlan and Hall, 2010).

Public data sets used in this study

ATAC-seq for XY mESCs is from GSM2247119, H3K27ac and H3K36me3 ChIP-seq in E14 cells is from mouse ENCODE ENCSR000CGQ and ENCSR253QPK respectively. 4sU-RNA-seq, PWWP2A, Pol Ser5, H3K9ac, and H3K27ac ChIP-seq are from GSE112114. DECAP-seq data for E14 and its derived Dnmt3b knockout are from GSE72854.

Supplemental References

Brocks, D., Schmidt, C.R., Daskalakis, M., Jang, H.S., Shah, N.M., Li, D., Li, J., Zhang, B., Hou, Y., Laudato, S., *et al.* (2017). DNMT and HDAC inhibitors induce cryptic transcription start sites encoded in long terminal repeats. Nat Genet *49*, 1052-1060.

Chen, K., Xi, Y., Pan, X., Li, Z., Kaestner, K., Tyler, J., Dent, S., He, X., and Li, W. (2013). DANPOS: dynamic analysis of nucleosome position and occupancy by sequencing. Genome Res *23*, 341-351.

Dobin, A., Davis, C.A., Schlesinger, F., Drenkow, J., Zaleski, C., Jha, S., Batut, P., Chaisson, M., and Gingeras, T.R. (2013). STAR: ultrafast universal RNA-seq aligner. Bioinformatics *29*, 15-21.

Haberle, V., Forrest, A.R., Hayashizaki, Y., Carninci, P., and Lenhard, B. (2015). CAGEr: precise TSS data retrieval and high-resolution promoterome mining for integrative analyses. Nucleic Acids Res *43*, e51.

Heinz, S., Benner, C., Spann, N., Bertolino, E., Lin, Y.C., Laslo, P., Cheng, J.X., Murre, C., Singh, H., and Glass, C.K. (2010). Simple combinations of lineage-determining transcription factors prime cis-regulatory elements required for macrophage and B cell identities. Mol Cell *38*, 576-589.

Li, H., Handsaker, B., Wysoker, A., Fennell, T., Ruan, J., Homer, N., Marth, G., Abecasis, G., and Durbin, R. (2009). The Sequence Alignment/Map format and SAMtools. Bioinformatics *25*, 2078-2079.

Neri, F., Rapelli, S., Krepelova, A., Incarnato, D., Parlato, C., Basile, G., Maldotti, M., Anselmi, F., and Oliviero, S. (2017). Intragenic DNA methylation prevents spurious transcription initiation. Nature *543*, 72-77.

Quinlan, A.R., and Hall, I.M. (2010). BEDTools: a flexible suite of utilities for comparing genomic features. Bioinformatics *26*, 841-842.

Raudvere, U., Kolberg, L., Kuzmin, I., Arak, T., Adler, P., Peterson, H., and Vilo, J. (2019). g:Profiler: a web server for functional enrichment analysis and conversions of gene lists (2019 update). Nucleic Acids Res *47*, W191-W198.

Zhang, T., Wei, G., Millard, C.J., Fischer, R., Konietzny, R., Kessler, B.M., Schwabe, J.W.R., and Brockdorff, N. (2018). A variant NuRD complex containing PWWP2A/B excludes MBD2/3 to regulate transcription at active genes. Nat Commun *9*, 3798.
